# Transitioning to Digital: A Single-Center Experience of Electronic Consent Implementation in General Surgery at a UK District General Hospital

**DOI:** 10.7759/cureus.104990

**Published:** 2026-03-10

**Authors:** Qasif Qavi, Rizwan Ahmed, Shashi Kumar, Firas Alkistawi, Abdalla Saad Abdalla Al-Zawi

**Affiliations:** 1 General Surgery, Basildon and Thurrock University Hospitals NHS Foundation Trust, Basildon, GBR; 2 Surgery, Princess Royal University Hospital, Orpington, GBR; 3 Surgery, Mid and South Essex NHS Foundation Trust, Basildon, GBR; 4 General Surgery, Mid and South Essex NHS Foundation Trust, Basildon, GBR; 5 General and Breast Surgery, Mid and South Essex NHS Foundation Trust, Basildon, GBR; 6 General and Breast Surgery, Basildon and Thurrock University Hospitals NHS Foundation Trust, Basildon, GBR; 7 General and Breast Surgery, Anglia Ruskin University, Chelmsford, GBR

**Keywords:** e-consent, emergent general surgery, healthcare digitalization, montgomery, patient consent

## Abstract

Introduction: Informed consent is an ethical and legal prerequisite for surgical intervention. In the United Kingdom, the Montgomery v Lanarkshire Health Board ruling shifted the standard toward a patient-centered model, necessitating a personalized discussion of "material risks". Traditional paper-based consent is often hampered by poor legibility and loss of physical records. This study evaluates a pilot electronic consent (e-consent) system in a general surgery department through clinician feedback.

Methods: A retrospective cross-sectional survey was conducted among 16 clinicians at Basildon University Hospital following the completion of a pilot phase for e-consent in appendicectomy and abscess procedures. Data on efficiency, hardware reliability, and compliance with Montgomery standards were collected via Likert-scale (1-5) responses.

Results: Clinicians reported that e-consent was faster than paper (mean 4.06/5) and intuitive to navigate (mean 4.13/5). Notably, clinicians reported high confidence in meeting Montgomery standards (mean 4.19/5). However, hardware availability (mean 3.25/5) and software reliability (mean 3.50/5) were identified as primary barriers. Despite these hurdles, 81.25% of participants preferred e-consent over traditional methods for future use.

Conclusion: E-consent improves the standardization and legal robustness of the consenting process. Institutional investment in hardware infrastructure is essential for sustainable hospital-wide adoption.

## Introduction

The legal framework for obtaining informed consent in the United Kingdom was fundamentally redefined by the landmark Montgomery v Lanarkshire Health Board ruling [1]. This shift moved surgical practice away from the paternalistic "Bolam" standard toward a patient-centered model, where clinicians must ensure patients are informed of any "material risks" and reasonable alternative treatments [2].

In the high-pressure environment of a district general hospital (DGH), traditional paper-based consent forms (Consent Form 1) are frequently associated with significant shortcomings. Studies have shown that paper forms are often plagued by illegibility, the inconsistent documentation of procedure-specific risks, and the logistical risk of being lost between the ward and the operating theater [3,4]. Such deficiencies not only compromise patient safety but also increase the medicolegal vulnerability of the Trust [5].

To address these challenges, Basildon University Hospital initiated a pilot rollout of an electronic consent (e-consent) system within the General Surgery department. The pilot focused on two high-volume emergency procedures: appendicectomy and incision and drainage (I&D) of abscesses. Digital systems offer the potential to improve documentation accuracy and patient comprehension through the use of standardized, procedure-specific templates [6]. This article evaluates the clinician-reported experience and the systemic barriers encountered during the initial implementation.

## Materials and methods

Study design and participants

We conducted a retrospective cross-sectional survey of clinicians who participated in the General Surgery e-consent pilot at Basildon University Hospital in Basildon, England. The survey was distributed following the initial pilot phase to capture a comprehensive overview of the implementation experience. The study analyzed responses from 16 surgical team members: two consultants, nine registrars, and five senior house officers.

Data collection

Participants evaluated the digital system using a structured 10-item questionnaire (see Appendices). Outcomes were measured on a 5-point Likert scale (1: strongly disagree to 5: strongly agree). The domains assessed included efficiency, usability, legal compliance, and infrastructure (hardware availability and software reliability). Because the study was conducted retrospectively, participants were asked to reflect on their total experience throughout the pilot duration.

The intervention

The e-consent platform utilized standardized templates pre-populated with "material risks". The system allowed for digital signatures on a mobile tablet, with the final document automatically integrating into the patient's electronic health record (EHR).

## Results

Demographics and clinician experience

The survey cohort comprised 16 clinicians with varying levels of seniority and hands-on experience with the digital system. The majority of respondents were registrars, and over 40% of the total cohort had completed more than 11 digital consents during the pilot period. The detailed breakdown of participant demographics and their utilization of the system is presented in Table 1.

**Table 1:** Participant demographics and experience (N=16) SHO: senior house officer; FY2: foundation year 2

Characteristic	Category	Count (n)	Percentage (%)
Designation	Registrar	9	56.25%
	SHO/FY2	5	31.25%
	Consultant	2	12.5%
Consents completed	1-5	4	25%
	6-10	5	31.25%
	11+	7	43.75%
Future preference	E-consent	13	81.25%
	No preference	3	18.75%

Quantitative system performance

The digital system was highly regarded for its clinical and legal utility, though infrastructure metrics were significantly lower. The highest mean score was recorded for confidence in meeting Montgomery standards, while hardware availability was identified as the primary bottleneck. The quantitative performance metrics are summarized in Table 2.

**Table 2:** Clinician evaluation of e-consent performance (N=16)

Domain	Mean score (1-5)	Standard deviation (SD)
Confidence in Montgomery standards	4.19	0.98
Easy to navigate	4.12	0.81
Faster than paper	4.06	0.93
Bedside consent effectiveness	3.88	0.89
Reliability of consent and hardware	3.5	1.1
Hardware availability	3.25	1.13

Qualitative analysis of challenges and recommendations

A thematic analysis of the qualitative feedback revealed significant technical and logistical friction. Clinicians frequently reported difficulty in locating available hardware and noted that the battery life of shared tablets was often insufficient for long shifts. Connectivity issues, including WiFi dead zones and internet slowness, led to software lag during the signature process. Some respondents also identified login issues and a lack of intuitive user interface (UI) customization as barriers to efficiency.

To facilitate a successful hospital-wide rollout, clinicians recommended the provision of dedicated, ward-based tablet lockers and charging stations. There was also a strong call for the installation of signal boosters to address poor connectivity and the implementation of Single Sign-On (SSO) integration to reduce administrative friction. Furthermore, the use of stylus pens was suggested to allow for more detailed anatomical diagrams during the consent discussion. Despite these challenges, 81.25% of clinicians favored e-consent, and no participants requested a return to paper forms.

## Discussion

Our findings demonstrate that digital consent systems significantly improve the confidence of doctors in the legibility and legal robustness of surgical documentation. The high confidence reported in meeting Montgomery standards (4.19/5) suggests that standardized templates act as an effective checklist for "material risks" that are frequently omitted in handwritten forms [1,3,6].

The infrastructure gap

The "hardware bottleneck" (3.25/5) identified in this study is a universal challenge in NHS digital transformations. As highlighted by the Topol Review, the success of digital interventions is contingent upon "digital readiness", which includes both technical infrastructure and seamless accessibility at the point of care [7]. Our data suggests that while the software is clinically superior, its effectiveness is currently capped by a lack of dedicated hardware and reliable hospital-wide WiFi.

Comparison with current literature

Our results align with quality improvement projects at other UK Trusts, which noted that digital consent significantly reduced "errors of omission" in emergency surgery [3,8]. Furthermore, micro-costing analyses suggest that while initial hardware costs are significant, the reduction in administrative errors and potential medicolegal payouts provides a positive long-term return on investment [9].

Limitations

This study is limited by its retrospective nature, which may introduce recall bias. The small sample size (n=16) in a single-center pilot also limits generalizability. Furthermore, the study focused on simple emergency procedures; the complexity of consenting for elective oncological or multi-stage procedures was not assessed. Future research should evaluate patient-reported experience measures (PREMs) to ensure digital forms improve comprehension.

## Conclusions

The e-consent pilot at Basildon Hospital has successfully enhanced clinician confidence in legal compliance and administrative efficiency. While clinician preference is strong (81.25%), sustainable hospital-wide adoption requires significant investment in robust hardware infrastructure, dedicated ward-based tablets, and seamless WiFi connectivity to ensure seamless connectivity. Further studies with larger groups and including patients in the research would help assess the benefit of e-consent in a more robust manner.

## Appendices

**Table 3:** Clinician experience questionnaire (electronic consent pilot) SHO: senior house officer; FY2: foundation year 2

Designation	No. of consents completed?	Faster than paper	Hardware availability	Confidence in Montgomery standards?	Bedside consent effectiveness	Reliability of consent and hardware	Easy to navigate	Biggest challenge encountered?	Recommendation for changes?	Future preference?	
SHO/FY2	11+	4	3	4	4	4	4	No tablet	More hardware	E-consent	
Registrar	11+	2	4	3	4	2	2	Slowness of the internet	Retraining	E-consent	
SHO/FY2	1-5	5	5	5	5	5	5	Chasing up the patient to fill out the consent form		E-consent	
Registrar	11+	5	2	5	2	2	4	-	-	E-consent	
Registrar	6-10	4	3	5	4	4	5	Availability of tablet and logins	More procedures	E-consent	
Registrar	1-5	5	5	5	5	5	4	-	-	E-consent	
SHO/FY2	6-10	4	2	2	3	2	4	Finding available hardware	Having a dedicated iPad for consent	No preference	
Registrar	11+	3	2	3	4	4	4	No tablets or poor connection	It should be used mainly in elective cases with a printout on the day of the surgery as a routine	E-consent	
Registrar	11+	4	3	5	3	4	4	Not always having the hardware on hand to consent; certain values available only as drop down and not free hand typing	Allow for more easily available hardware for e-consenting particular on-call shifts. Allow for more freedom to input personalized data and requests	No preference	
Consultant	6-10	4	4	5	4	3	5	Battery life of tablets	Charging stations in theaters	E-consent	
Registrar	1-5	5	2	4	5	4	4	Login issues	Single sign-on integration	E-consent	
SHO/FY2	11+	3	3	3	3	2	3	Software lag	Optimized mobile app	E-consent	
Registrar	6-10	5	5	5	5	5	5	-	Rollout to all departments	E-consent	
Consultant	11+	5	4	5	4	4	5	WiFi dead zones in some wards	Install more signal boosters	E-consent	
SHO/FY2	6-10	3	3	4	3	3	4	Small screen for complex diagrams	Provision of stylus pens	E-consent	
Registrar	1-5	4	2	4	4	3	4	Difficulty locating the tablet cart	Dedicated lockers in each ward	No preference	
